# Atlanta Medical College

**Published:** 1857-08

**Authors:** 


### EDITORIAL AND MISCELLANEOUS.

### ATLANTA MEDICAL COLLEGE.

We would desire to inform those who feel an interest in the success of this Institution, that, notwithstanding the extraordinary efforts, which were made in several quarters, previous to the commencement of the present Session, to prejudice the public mind unfavorably, in reference to the time selected for the Course of Lectures, we are enabled to report a *decided* progress in *every* particular.

In the number of the Class there has been a very considerable increase, most remarkable, when taken into connection with the uncertainty which prevailed as to the ability of the Faculty to accomplish, unaided, the task of erecting suitable buildings, and furnishing the other necessary appliances for a first class College; and especially when taken into connection with the energetic labors of rival institutions to pull us down.

So long as the patronage of *certain* Southern Schools was not interfered with by the establishment of a new institution, at a point where it was found to be perfectly practicable to give a full, thorough, and last, though not least, a *successful* Course of Medical Lectures in the *summer*, no word of complaint is heard—judging by their own localities, on the banks of tadpole rivers, or in the vicinity of swampy rice fields, subject to the visitation of those deadly pestilences, Cholera and Yellow Fever, and besides, imagining that "they were the people, and wisdom would die with them"—that anywhere else than certain would-be great cities, was "no where"—they had no fear of the success of any such movement in the South; but by degrees, becoming aroused to the knowledge of the fact that there was *one* locality, South of Mason and Dixon's Line, which furnished the facilities for successful *Summer* teaching, and that the effort to use it as such, was no longer an experiment, it was apparently resolved by one or two of the "regulators" of medical affairs, that this shall not be—medicine shall not be taught in the *South* in the *Summer*,—indeed there are only certain points where it ought to be taught at all; we have those points, *therefore*, we ought to do and will do all the medical teaching, if we can. It remains to be seen whether this logic will find a response of approval, either from "Americanism" or "Democracy" in medical ranks. Of one thing, certain puffed up gentlemen may be assured, the masses of the profession will never countenance an "Aristocracy" in Medicine, even though it may be of the codfish order.

In this connection, it affords us *pleasure* to acknowledge the friendly demonstrations of most of the Colleges, North and South, with whom we have had any intercourse, and it affords us *special pleasure*, to acknowledge the many obligations conferred upon us, by individual members of the profession, scattered all over the land, who have come up to our help in so many ways, and to whom we owe a debt of gratitude that will at least be held in remembrance, if never discharged.

